# Mediating effects of self-stigma and depression on the association between autistic symptoms and recovery in patients with schizophrenia-spectrum disorders: a cross-sectional study

**DOI:** 10.1186/s12888-021-03472-z

**Published:** 2021-09-23

**Authors:** Hiroshi Komatsu, Takashi Ono, Goh Onoguchi, Hiroaki Tomita, Yoshihisa Kakuto

**Affiliations:** 1 Department of Psychiatry, Tohoku University Hospital, 1-1 Seiryo-machi, Aobaku, Sendai, 980-8573 Japan; 2 Department of Psychiatry, Miyagi Psychiatric Center, Mubanchi, Tekurada, Natori, 981-1231 Japan; 3 Department of Psychiatry, Graduate School of Medicine, Tohoku University, Sendai, Japan; 4 Department of Disaster Psychiatry, International Research Institute of Disaster Science, Tohoku University, Sendai, Japan; 5 Tohoku Medical Megabank Organization, Tohoku University, Sendai, Japan; 6 Department of Disaster Psychiatry, Graduate School of Medicine, Tohoku University, Sendai, Japan

**Keywords:** Autistic symptoms, Depression, Schizophrenia-spectrum disorders, Self-stigma, Structural equation modeling

## Abstract

**Background:**

Several studies have indicated that self-stigma is associated with depressive symptoms and could be a barrier to recovery in patients with schizophrenia-spectrum disorders. More recently, an association between autistic symptoms and self-stigma was found in schizophrenia-spectrum patients. This study aimed to investigate the association between self-stigma, autistic and depressive symptoms, and recovery in patients with schizophrenia.

**Methods:**

In total, 105 participants were evaluated using the Autism Spectrum Quotient, the Internalized Stigma of Mental Illness Scale, the Quick Inventory of Depressive Symptomatology, and the Recovery Assessment Scale to investigate autistic symptoms, self-stigma, depressive symptoms, and recovery, respectively. The relationship between self-stigma, autistic symptoms, depressive symptoms, and recovery was assessed using structural equation modeling analysis.

**Results:**

Impaired attention switching, one symptom of autism, was found to positively affect stereotype endorsement, which negatively influenced recovery through depressive symptoms. Moreover, problems with communication skills negatively affected recovery through depressive symptoms. Concerning self-stigma, stereotype endorsement and perceived discrimination had a negative effect on recovery through depressive symptoms, whereas stigma resistance had a direct negative effect on recovery.

**Conclusions:**

This study may provide meaningful insight into the psychological structure of recovery and could inform effective interventions for patients with schizophrenia-spectrum disorders. This was a cross-sectionally designed study; therefore, further longitudinal studies are needed to identify the causal relationships between self-stigma, autistic and depressive symptoms, and recovery.

**Supplementary Information:**

The online version contains supplementary material available at 10.1186/s12888-021-03472-z.

## Background

Prior investigations have indicated that the stigma related to patients with schizophrenia is a global problem [1]. Patients with serious mental illnesses, such as schizophrenia, have been reported to encounter different forms of stigmatization [2, 3]. Corrigan et al. categorized these types of stigma into “public stigma,” “self-stigma,” and “label avoidance” [4]. Self-stigma arises when people with psychiatric disorders who live in a society that endorses stigmatizing ideas internalize such ideas and believe that they are less valuable because they have a mental illness [5]. Watson et al. hypothesized that self-stigma develops through three sequential processes: stereotype awareness, stereotype agreement, and self-concurrence [6]. Self-stigma has a negative effect on patients with mental illness (e.g., low self-esteem and self-efficacy, reduced quality of life (QOL), and depressive symptoms) [6–12]. Moreover, self-stigma can also be a barrier to recovery and negatively influence treatment adherence [13–17].

In a recent meta-analysis, higher levels of autistic symptoms in patients with schizophrenia-spectrum disorders were identified compared with healthy controls [18]. Furthermore, higher levels of autistic traits have recently been associated with poorer social functioning in patients with psychosis, and autism spectrum traits have been found to be predictors of 1-year poor recovery outcomes in patients with a first-episode psychosis [19, 20].

Exploring the hypothesis that patients with schizophrenia-spectrum disorders exhibiting a higher level of autistic symptoms would have difficulty in coping with stigmatization due to reduced communication skills and cognitive flexibility and would be more likely to internalize stigma than those with fewer autistic symptoms, our recent investigation revealed a significant association between autistic symptoms and self-stigma in patients with schizophrenia-spectrum disorders [21]. Considering that self-stigma is associated with depressive symptoms and recovery, our previous findings suggested that autistic symptoms could also directly or indirectly correlate with the other three factors.

Elucidation of how self-stigma and autistic and depressive symptoms affect recovery may provide meaningful insight into the psychological structure of recovery and help inform effective interventions for recovery in patients with schizophrenia-spectrum disorders. Therefore, this study aimed to better elucidate the relationship between autistic and depressive symptoms, self-stigma, and recovery in patients with schizophrenia-spectrum disorders using structural equation modeling (SEM) analysis.

## Methods

### Participants

We recruited 110 patients at the Miyagi Psychiatric Center with schizophrenia-spectrum disorders from March 2019 to November 2019 to assess their symptoms of autism, self-stigma, and depression, as well as the extent of recovery. Psychological assessment data were collected from each patient according to a method described in a previous study [21]. All psychological data were successfully obtained from 105 patients (three patients withdrew consent and two did not complete the questionnaires). Two psychiatrists diagnosed participants with schizophrenia (*n* = 102), schizoaffective disorder (*n* = 2), or delusional disorder (*n* = 1) using the Diagnostic and Statistical Manual of Mental Disorders Fifth Edition (DSM-5). We excluded patients with intellectual disabilities or organic psychiatric disorders as well as those aged > 20 years. Furthermore, patients with comorbid substance use disorders were excluded because of the effect of comorbidities on depressive symptoms. During the participant recruitment process, the researcher fully explained the contents of this study to each inpatient and outpatient in the acute care ward. Written informed consent was obtained from each participant. Among the inpatients, those who were assessed by the physician as having improved acute psychiatric symptoms and who were permitted to participate in the study were included. The duration of hospitalization at the time of evaluation (± standard deviation [SD]) was 53.0 ± 61 days (Table 1), and only 1 of 50 patients had been hospitalized for > 6 months.

**Table 1:** Patient characteristics

	Total samples (*N* = 105)	Outpatients (*N* = 55, 52.3%)	Inpatients (*N* = 50, 47.7%)	*p-*value
Sex (male/female)^a^	48/57	22/33	26/24	0.244
Age (mean ± SD) ^b^	47.0 ± 13.3	47.8 ± 14.2	46.1 ± 12.4	0.529
Diagnosis^a^				
Schizophrenia	103	54	48	1
Schizoaffective disorder	2	0	2	
Delusional disorder	1	1	0	
Age of onset (mean ± SD) ^b^	28.8 ± 11.5	30.2 ± 11.7	27.3 ± 11.1	0.156
Duration of hospitalization (at time of assessment, mean ± SD, days)	53.0 ± 66.0		53.0 ± 66.0	
Number of hospitalizations ^b^	4.0 ± 5.1	3.5 ± 4.7	4.7 ± 5.5	0.528
Years of education^a^				0.912
≤ 9	36	18	18	
> 9- ≤ 12	36	20	16	
> 12	32	17	15	
Marital state^a^				
Single	71	34	37	0.372
Married	20	13	7	
Divorced	14	8	6	
Widow	1	1	0	
Partners^a^				
Yes	84	45	39	0.635
No	21	10	11	
Antipsychotic medication^b^ Antipsychotic dose (SGA + FGA) (CP equivalent, mean ± SD, mg/day)	617.2 ± 349.9	558.1 ± 360.0	682.2 ± 323.0	0.67
Antipsychotic dose of SGA (CP equivalent, mean ± SD, mg/day)	582.8 ± 337.5	533.2 ± 352.8	637.4 ± 316.7	0.114
Antipsychotic dose of FGA (CP equivalent, mean ± SD, mg/day)	34.9 ± 108.23	24.9 ± 84.6	44.8 ± 129.5	0.351

^a^ Fisher’s exact test *p*-value for the difference in sex, diagnosis, years of education, marital status, and partners between the two groups

^b^ Unpaired *t*-test *p*-value for the difference in age between the two groups

*AQ* Autism-Spectrum Quotient, *CP C*hlorpromazine, *FGA* First-generation antipsychotics, *IMSI* Internalized Stigma of Mental Illness, *QIDS,* Quick Inventory of Depressive Symptomatology, *RAS* Recovery Assessment Scale, *SGA* Second-generation antipsychotics

This study was conducted in accordance with the principles outlined in the Declaration of Helsinki and was approved by the Ethics Committee of the Miyagi Psychiatric Center (MPC-20190320). After the study procedures had been explained to the participants, written informed consent was obtained in accordance with the Declaration of Helsinki.

### Assessment of self-stigma

We assessed self-stigma using the Internalized Stigma of Mental Illness Scale, Japanese version (ISMI-J), which is a self-reported scale developed by Ritsher et al. [11]. The ISMI is a commonly used scale to assess self-stigma in various countries. A Japanese version has been prepared and validated by Tanabe et al. [22]. The ISMI consists of 29 items assessed using a 4-point Likert scale ranging from 1. “I do not think so” to 4. “I very much think so,” and five subscales (alienation [1, 5, 8, 16, 17, 21], stereotype endorsement [2, 6, 10, 18, 19, 23, 24], perceived discrimination [3, 15, 22, 25, 26], social withdrawal [4, 9, 11–13, 20], and stigma resistance [7, 14, 27–29]). The average ISMI score is interpreted as follows: 1.00–2.00, no or very mild self-stigma; 2.01–2.50, a mild level of self-stigma; 2.51–3.00, a moderate level of self-stigma; and 3.01–4.00, a severe level of self-stigma. The internal consistency (Cronbach’s α) for the ISMI-J was 0.93 in this study.

### Assessment of autistic symptoms

We evaluated autistic symptoms in patients with schizophrenia-spectrum disorders using the Autism-Spectrum Quotient, Japanese version (AQ-J). Baron-Cohen et al. developed the AQ [23]. A Japanese version of the AQ-J has been prepared and validated by Wakabayashi et al. [27]. The AQ is a 50-item self-report scale divided into five subscales: social skills, attention switching, attention to detail, communication skills, and imagination. Each item is rated on a 4-point Likert scale with the following response options: “definitely agree,” “slightly agree,” “slightly disagree,” and “definitely disagree.” Of these, “definitely agree” and “slightly agree” are scored as “1,” and “slightly disagree” and “definitely disagree” are scored as “0” in 24 items (No. 2, 4–7, 9, 12, 13, 16, 18–23, 26, 33, 35, 39, 41–43, 45, and 46). The Cronbach’s α for the AQ-J was 0.72 in this study.

### Assessment of depressive symptoms

Depressive symptoms were evaluated using the Quick Inventory of Depressive Symptomatology, Japanese version (QIDS-J) [25]. The QIDS is a 16-item self-report scale developed by Rush et al. [28]. A Japanese version (QIDS-J) has been prepared and validated by Fujisawa et al. [25]. The higher the scores of the QUID score, the more severe the depressive symptoms. The Cronbach’s α was 0.84 in this study.

### Assessment of recovery

The recovery level was evaluated using the Recovery Assessment Scale, Japanese version (RAS-J). The RAS is a 24-item self-reported scale [29], with each item rated on a 5-point Likert scale with response options ranging from: 1. “I do not think so at all” to 5. “I think so”. A Japanese version of the RAS (RAS-J) has been prepared and validated by Chiba et al. [26]. The higher the QIDS scores, the higher the recovery level. Cronbach’s α was 0.930 in this study.

### Statistical analysis

Statistical analyses were performed using SPSS Statistics 26 and Amos 26 (Japan IBM, Tokyo, Japan) software. We categorized the participants into two groups (outpatients and inpatients).

The demographic variables among each group were compared using a Fisher’s exact test and an unpaired t-test, as appropriate.

The correlation between demographic variables and each scale was analyzed using Spearman’s correlation analysis. Based on the results of Spearman’s correlation analysis, we constructed a hypothetical initial model regarding the relationship between self-stigma, autistic and depressive symptoms, and recovery. We removed the paths with *p*-values > 0.05 from each initial model and examined whether the model fit improved. The best-fitting path model was ultimately adopted in the SEM analysis.

We performed SEM analysis to assess the goodness-of-fit of the hypothetical model. The SEM was performed using SPSS Amos 23 software. Model fits were estimated using the maximum likelihood method. Chi-square statistics were used to test the goodness-of-fit model. The following fit indices were calculated: goodness-of-fit index (GFI), adjusted GFI (AGFI), comparative fit index (CFI), Akaike information criterion (AIC), and root mean square error of approximation (RMSEA). Bonferroni corrections were applied for post-hoc multiple comparison tests. Statistical significance was defined as a two-tailed *p*-value of < 0.05.

## Results

### Patient characteristics

The mean age (±SD) was 47.0 ± 13.3 years. No significant differences were observed in terms of sex, age, diagnosis, age at onset, number of hospitalizations, education history, marital status, partnership, and antipsychotic dose among outpatients (*N* = 55) and inpatients (*N* = 50) (Table 1). Although the inpatients scored higher in “attention to detail” (one of the AQ subscales) than the outpatients (unpaired t-test, *p* = 0.021; Table 2), there was no significant difference in the ISMI, RAS, and QIDS scores between outpatients and inpatients (Table 2).

**Table 2:** The difference in the ISMI, the AQ, the QIDS, and the RAS score between outpatients and inpatients

	Total samples (*N* = 105)	Outpatients (*N* = 55, 52.3%)	Inpatients (*N* = 50, 47.7%)	*p*-value
ISMI total score	2.2 ± 0.5	2.2 ± 0.5	2.2 ± 0.5	0.515
Alienation	2.3 ± 0.8	2.3 ± 0.78	2.3 ± 0.8	0.455
Stereotype endorsement	2.0 ± 0.6	2.0 ± 0.6	2.0 ± 0.6	0.608
Perceived discrimination	2.1 ± 0.6	2.1 ± 0.6	2.1 ± 0.6	0.556
Social withdrawal	2.2 ± 0.7	2.3 ± 0.7	2.1 ± 0.7	0.412
Stigma resistance	2.7 ± 0.5	2.7 ± 0.5	2.8 ± 0.5	0.633
AQ total score	23.9 ± 6.4	23.1 ± 6.5	24.7 ± 6.3	0.161
Social skills	4.8 ± 2.4	4.7 ± 2.4	5.0 ± 2.3	0.4
Attention switching	5.3 ± 2.1	5.1 ± 1.8	5.5 ± 1.9	0.171
Attention to detail	5.3 ± 2.1	4.8 ± 2.2	5.8 ± 1.9	0.021*
Communication skills	4.1 ± 2.3	4.0 ± 2.2	4.1 ± 2.7	0.756
Imagination	4.4 ± 2.0	4.4 ± 1.9	4.4 ± 2.2	0.992
QIDS	10.4 ± 6.4	10.2 ± 6.9	10.5 ± 5.8	0.55
RAS	78.8 ± 18.2	79.3 ± 18.7	78.3 ± 17.9	0.557

The table shows unpaired t-test p-value for the difference in age between the three groups

Bold values indicate statistical significance at *p* < 0.05 level

*p < 0.05

*AQ* Autism-Spectrum Quotient, *IMSI* Internalized Stigma of Mental Illness, *QIDS* Quick Inventory of Depressive Symptomatology, *RAS* Recovery Assessment Scale

### Bivariate correlations between self-stigma, autistic and depressive symptoms, recovery, and patient characteristics

There was a significant negative correlation between the AQ total score and the QIDS score and the onset of age in patients with schizophrenia-spectrum disorders (*r* = − 0.34, *p* < 0.05 and *r* = − 0.349, *p* < 0.05, respectively; Table 3). Spearman’s correlation analysis revealed that the AQ total score was positively correlated with the ISMI total score (*r* = 0.475, *p* < 0.001), the ISMI “alienation” subscale (*r* = 0.393, *p* < 0.01), the ISMI “stereotype endorsement” subscale (*r* = 0.424, *p* < 0.001), the “perceived discrimination” subscale (*r* = 0.465, *p* < 0.001), the “social withdrawal” subscale (*r* = 0.413, *p* < 0.001), and the QIDS score (*r* = 0.554, *p* < 0.001) in the patients (Table 4). “Attention switching,” a subscale of AQ, was positively correlated with the ISMI total score (*r* = 0.385, *p* < 0.01), the ISMI “alienation” subscale (*r* = 0.373, *p* < 0.01), the “stereotype endorsement” subscale (*r* = 0.366, *p* < 0.01), the “social withdrawal” subscale (*r* = 0.346, *p* < 0.05), and the QIDS score (*r* = 0.474, *p* < 0.001; Table 4). Additionally, positive correlations between the AQ “communication skills” subscale and the ISMI total score (*r* = 0.345, *p* < 0.05), the ISMI “perceived discrimination” subscale (*r* = 0.411, *p* < 0.001), and the QIDS score (*r* = 0.505, *p* < 0.001) were observed (Table 4). Further, the QIDS score was positively associated with the ISMI total score, as well as the ISMI subscale, except for the “stigma resistance” subscale (*r* = 0.558 [ISMI “alienation” subscale] to 0.650 [ISMI total score], all *p* < 0.001; Table 4). In contrast, the RAS score was negatively associated with the ISMI total score (*r* = − 0.574, *p* < 0.001), all the ISMI subscales (*r* = − 0.516 [“stereotype endorsement” subscale] to − 0.385 [“stigma resistance” subscale], all *p* < 0.01), the AQ total score (*r* = − 0.462, *p* < 0.001), the AQ “social skills” subscale (*r* = − 0.458, *p* < 0.001), and the QIDS score (*r* = − 0.560, *p* < 0.001; Table 4).

**Table 3:** Bivariate correlations between self-stigma, autistic and depressive symptoms, and recovery with patient characteristics

	Age	Age of onset	Number of hospitalizations	Years of education	Antipsychotci dose
ISMI total score	−0.069	− 0.151	0.017	0.088	0.105
Alienation	−0.147	−0.176	0.006	0.067	0.086
Stereotype endorsement	0.041	−0.136	0.081	0.077	0.162
Perceived discrimination	−0.119	−0.223	0.046	0.078	0.131
Social withdrawal	−0.078	−0.106	0.021	0.044	0.118
Stigma resistance	0.024	0.088	0.02	0.017	−0.104
AQ total score	−0.171	− 0.34*	0.005	− 0.03	0.268
Social skills	−0.085	−0.201	− 0.206	−0.119	0.04
Attention switching	−0.068	−0.309	0.076	−0.038	0.261
Attention to detail	−0.088	−0.167	0.214	−0.022	0.09
Communication skills	−0.244	−0.272	− 0.064	−0.008	0.217
Imagination	−0.062	−0.116	0.012	0.054	0.169
QIDS	−0.073	−0.349*	0.084	−0.012	0.267
RAS	−0.007	0.124	−0.049	−0.032	− 0.068

The data show Spearman’s rank correlation coefficients

Bold values indicate statistical significance at *p* < 0.05 level

*p < 0.05

*AQ* Autism-Spectrum Quotient, *IMSI* Internalized Stigma of Mental Illness, *QIDS* Quick Inventory of Depressive Symptomatology, *RAS* Recovery Assessment Scale

**Table 4:** Differences in Spearman’s correlation coefficients between self-stigma, autistic and depressive symptoms, and recovery

	ISMI total score	Alienation	Stereotype endorsement	Perceived discrimination	Social withdrawal	Stigma resistance	QIDS	RAS
AQ total score	0.475***	0.393**	0.424***	0.465***	0.413***	0.086	0.554***	−0.462***
Social skills	0.312	0.207	0.242	0.261	0.247	0.252	0.306	−0.458***
Attention switching	0.385**	0.373**	0.366**	0.325	0.346*	0.009	0.474***	−0.295
Attention to detail	0.114	0.133	0.087	0.136	0.137	−0.092	0.069	0.041
Communication skills	0.345*	0.253	0.277	0.411***	0.303	0.036	0.505***	−0.304
Imagination	0.252	0.21	0.286	0.246	0.182	0.06	0.307	−0.321
QIDS	0.65***	0.558***	0.596***	0.659***	0.597***	0.006		−0.56***
RAS	−0.574***	−0.422***	− 0.516***	−0.475***	− 0.431***	−0.385**	− 0.56***	

The data show Spearman’s rank correlation coefficients

Bold values indicate statistical significance at *p* < 0.05 level

*p < 0.05, ** *p* < 0.01, ***p < 0.001

*AQ* Autism-Spectrum Quotient, *IMSI* Internalized Stigma of Mental Illness, *QIDS* Quick Inventory of Depressive Symptomatology, *RAS* Recovery Assessment Scale

### Structural equation modeling analysis of the relationship between self-stigma, autistic and depressive symptoms, and recovery

We hypothesized that patients with autistic symptoms would have difficulty in coping with stigmatization, more depressive symptoms, have slower recovery due to lower communication and social skills as well as reduced cognitive flexibility, and would be more likely to internalize stigma than those with fewer autistic symptoms. Ritsher et al. previously reported that self-stigma predicts depressive symptoms [24]. Using SEM analysis, Lien et al. showed that self-stigma mediates the association between insight and depressive symptoms [26]. A previous longitudinal study showed that more self-stigma at baseline was associated with a significant decrease in recovery after 1 year. In that study, an increase in self-stigma from baseline to follow-up predicted less recovery 1 and 2 years later [13].

**Fig. 1:**
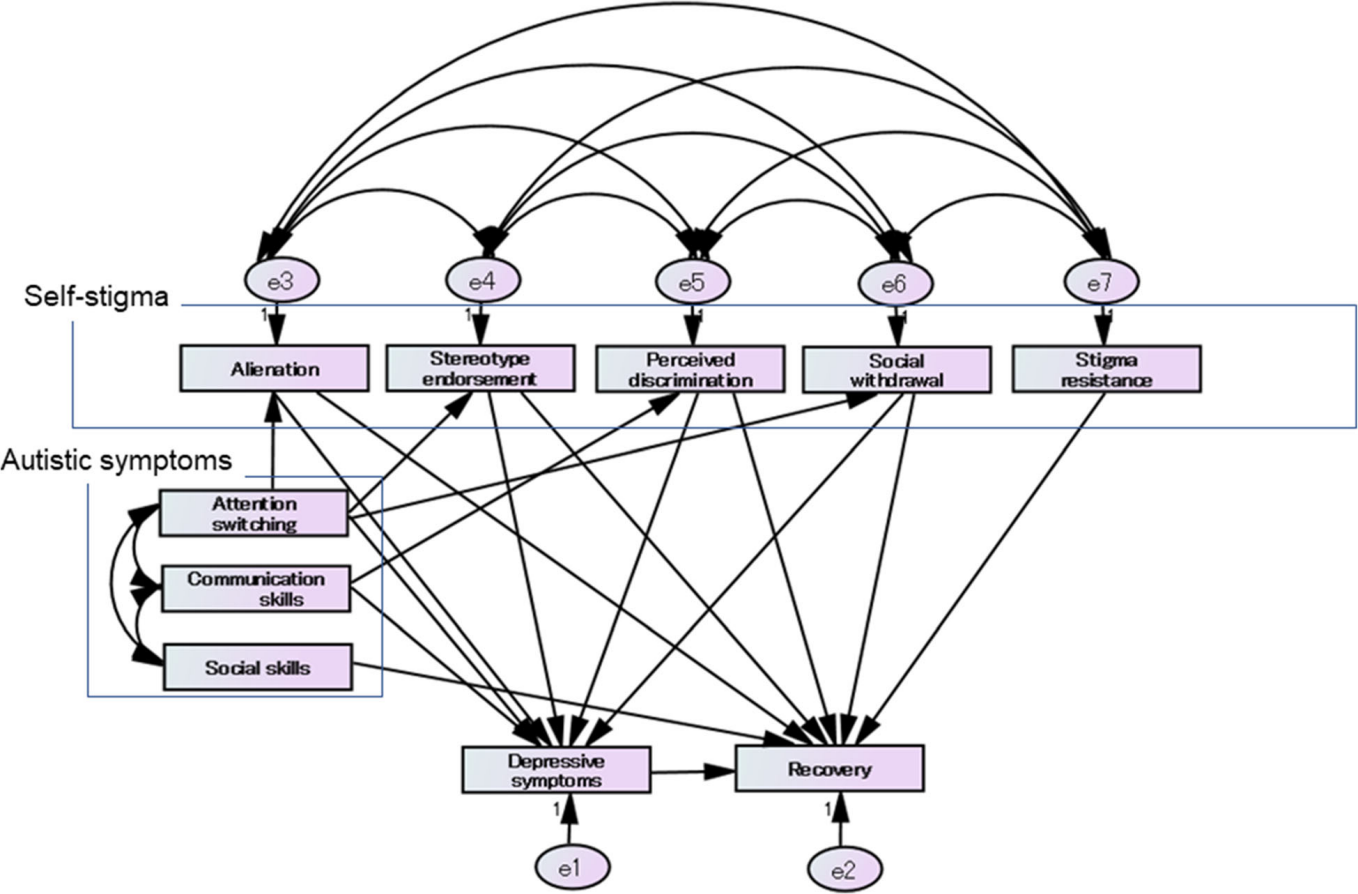
A hypothetical initial model showing the relationship between self-stigma, autistic and depressive symptoms, and recovery. The hypothetical initial models that are shown here assume that “attention switching,” a symptom of autism, affects “alienation,” “stereotype endorsement,” and “social withdrawal” of self-stigma, and depressive symptoms. “Communication skills,” a symptom of autism, is hypothesized to influence “perceived discrimination” and depressive symptoms. Further, in the initial model, we hypothesized that “social skills,” a symptom of autism, affects recovery in one way. Based on several previous studies and bivariate correlation analysis, we assumed that all subscales of self-stigma affect recovery and four subscales of self-stigma, except for “stigma resistance” influence depressive symptoms. The initial hypothetical model provided the following statistics for the model fit: chi-square statistic = 21.737, df = 16, *p* = 0.152; GFI = 0.962; AGFI = 0.868; CFI = 0.990; AIC = 99.737; and RMSEA = 0.059. Paths with *p*-values > 0.05 were removed from the initial models, and the model goodness-of-fit was reanalyzed

Based on Spearman’s correlation analysis results and based on our hypothesis and previous studies [13, 24, 30], we constructed a hypothetical initial model, as shown in Fig. 1.

We examined the goodness-of-fit of the hypothetical initial model using SEM analysis. The initial model statistics for the model fit were as follows: chi-square statistic = 21.737, df = 16, *p* = 0.152; GFI = 0.962; AGFI = 0.868; CFI = 0.990; AIC = 99.737; and RMSEA = 0.059.

**Fig. 2:**
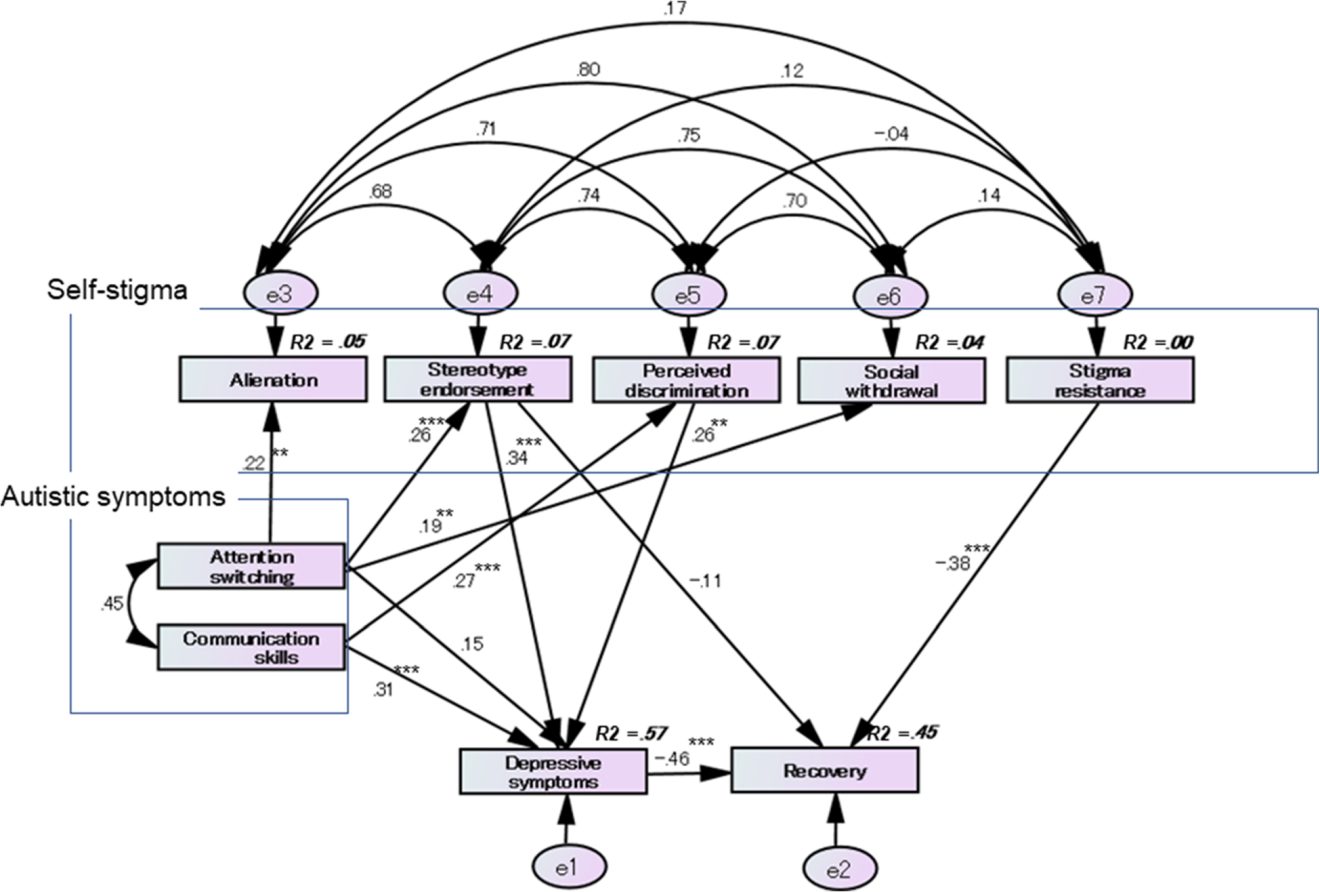
The best-fitting model for the relationship between self-stigma, autistic and depressive symptoms, and recovery. The adopted best-fitting model is shown in Fig. 2. The best-fitting model showed the following statistics for the model fit: chi-square statistic = 10.790, df = 14, *p* = 0.702; GFI = 0.978; AGFI = 0.929; CFI = 1.000; AIC = 72.790; and RMSEA < 0.001. The AQ “attention switching” subscale has a significant direct effect on the ISMI “alienation” subscale (SDE = 0.215, *p* = 0.001), “stereotype endorsement” subscale (SDE = 0.261, *p* < 0.001), and “social withdrawal” subscale (SDE = 0.191, *p* = 0.006). The AQ “communication skills” subscale has a significant direct effect on the ISMI “perceived discrimination” subscale (SDE = 0.270, *p* < 0.001) and the QIDS score (depressive symptoms) (SDE = 0.307, *p* < 0.001). The ISMI “stereotype endorsement” subscale directly affected the QIDS score (SDE = 0.343, *p* < 0.001). Further, the RAS score (recovery) was directly influenced by the “stigma resistance” subscale (SDE = − 0.377, *p* < 0.001) and the QIDS score (SDE = − 0.460, *p* < 0.001). The ISMI “stereotype endorsement” subscale (SIDE = − 0.158) and the AQ “communication skills” subscale (SIDE = − 0.174) indirectly affect the RAS score via the QIDS score

We removed the paths with *p*-values > 0.05 from each initial model and examined whether the model fit improved. We ultimately adopted the best-fitting model, as shown in Fig. 2. The best-fitting model statistics for the model fit were as follows: chi-square statistic = 10.790, df = 14, *p* = 0.702, GFI = 0.978; AGFI = 0.929; CFI = 1.000; AIC = 72.790, and RMSEA < 0.001.

The AQ “attention switching” subscale had a significant direct effect on the ISMI “alienation” subscale (standardized direct effect [SDE] = 0.215, *p* = 0.001), the “stereotype endorsement” subscale (SDE = 0.261, *p* < 0.001), and the “social withdrawal” subscale (SDE = 0.191, *p* = 0.006). The AQ “communication skills” subscale had a significant direct effect on the ISMI “perceived discrimination” subscale (SDE = 0.270, *p* < 0.001) and the QIDS score (depressive symptoms) (SDE = 0.307, *p* < 0.001). The ISMI “stereotype endorsement” subscale directly affected the QIDS score (SDE = 0.343, *p* < 0.001). Further, the RAS score (recovery) was directly influenced by the ISMI “stigma resistance” subscale and the QIDS score (SDE = − 0.377, *p* < 0.001 and SDE = − 0.460, *p* < 0.001, respectively). In addition, the ISMI “stereotype endorsement” subscale and the AQ “communication skills” subscale indirectly affected the RAS score via the QIDS score (standardized indirect effect [SIDE] = − 0.158 and SIDE = − 0.174, respectively).

## Discussion

One previous study revealed a significant association between autistic symptoms and self-stigma in patients with schizophrenia-spectrum disorders [21]. Considering that prior studies have shown that self-stigma is associated with depressive symptoms and recovery, we hypothesized that autistic symptoms would also directly or indirectly correlate with self-stigma, depressive symptoms, and recovery. To verify this hypothesis, this study investigated the association between self-stigma, autistic and depressive symptoms, and recovery in patients with schizophrenia-spectrum disorders using SEM analysis. The results of this study indicated that impaired attention switching, one symptom of autism, influenced recovery negatively through depressive symptoms and stereotype endorsement (a component of self-stigma). Moreover, problems with communication skills, a symptom of autism, negatively affected recovery via depressive symptoms. Of the self-stigma components, stereotype endorsement negatively affected recovery through depressive symptoms. In contrast, only stigma resistance had a direct negative effect on recovery.

In this study, a significant negative correlation between the total AQ score and age at onset was observed. One previous report showed that the age of onset was lower in patients with schizophrenia-spectrum disorders who had autism-spectrum disorders than in those without such disorders [31]. The results of this study indicated that a younger age of onset among patients with schizophrenia-spectrum disorders was associated with a higher AQ total score, which is consistent with the results of previous studies [21, 31]. We identified a weak positive correlation between age at onset and the QIDS score in the bivariate correlation analysis. However, as no definite association between age at onset and depressive symptoms has been previously reported in patients with schizophrenia-spectrum disorders, age at onset may be a confounding factor for the QIDS score. Hence, further verification of our finding is necessary in future.

An excessive D2 receptor blockade of antipsychotic drugs is known to cause side-effects such as dysphoria associated with depression [32]. Therefore, we analyzed the correlation between the chlorpromazine equivalents of antipsychotics and depressive symptoms, autistic symptoms, self-stigma, and recovery; however, no significant correlation was found.

Spearman’s correlation analysis showed that the AQ total score was positively correlated with the ISMI total score, the ISMI “alienation” subscale, the “stereotype endorsement” subscale, the “perceived discrimination” subscale, the “social withdrawal” subscale, and the QIDS score in patients with schizophrenia-spectrum disorders. We hypothesized that patients with autistic symptoms would have difficulty in coping with stigmatization, have more depressive symptoms due to more limited communication skills and have reduced cognitive flexibility, and would be more likely to internalize stigma than those with fewer autistic symptoms [21].

Therefore, we constructed a hypothetical model of the association between autistic symptoms and self-stigma and depressive symptoms, as shown in Fig. 2. Our hypothesis was supported by the SEM analysis. Difficulty in switching attention affected alienation, stereotype endorsement, and social withdrawal. However, difficulty in communication affected perceived discrimination and depressive symptoms. In contrast, the total AQ score was negatively associated with the RAS score in our study participants. Of the AQ subscales, only social skills were found to have a significant negative correlation with the RAS score. However, in the SEM analysis, social skills did not directly affect recovery.

Bivariate correlation analysis showed a positive association between the QIDS score and the ISMI total score, as well as the ISMI subscale, except for the “stigma resistance” subscale. In contrast, the RAS score was negatively associated with the ISMI total score, all the ISMI subscales, and the QIDS score. One previous study reported that self-stigma predicts depressive symptoms [24]. A longitudinal study showed that self-stigma was strongly correlated with depression over time, whereby higher scores of self-stigma were associated with higher depression [33]. As previously mentioned, Oexle et al. reported in a longitudinal study that an increase in self-stigma from baseline predicted less recovery 1 and 2 years later [13]. Therefore, we constructed a hypothetical model to determine the correlation of self-stigma with depressive symptoms and recovery, as shown in Fig. 2. SEM analysis indicated that stereotype endorsement as part of self-stigma directly affected depressive symptoms. Furthermore, stigma resistance and depressive symptoms directly influenced recovery. Stereotype endorsement and difficulty in communication skills indirectly affected recovery via depressive symptoms. Several recent studies have shown autistic traits in patients with psychotic disorders, including first-episode psychosis, which has been associated with poor recovery outcomes and social functioning [19, 20, 34]. Considering these previous findings and the results of our study, self-stigma and depression may partially be related to the association between high levels of autistic traits in patients with psychosis and poor recovery-related outcomes and social functioning.

Our study results suggest that self-stigma and depressive symptoms mediate the association between autistic symptoms and recovery in patients with schizophrenia-spectrum disorders. Therefore, the results highlight the importance of careful assessment and intervention for self-stigma and depressive symptoms in patients with schizophrenia-spectrum disorders who exhibit more autistic symptoms.

This study had several limitations. First, this study could not determine a causal relationship between self-stigma, autistic and depressive symptoms, and recovery because it was a cross-sectional study. Second, the results cannot be generalized to the general population, as the data were obtained from a single medical institution. Third, since we used self-report rating scales in the study, the scores may have been affected by participants’ mental statuses at the times of evaluation. AQ scores may be affected not only by depressive symptoms but also by the intensity of anxiety and negative symptoms [18, 35]. However, in this study, we did not assess psychiatric symptoms other than depression using the Brief Psychiatric Rating Scale (BPRS) or the Positive and Negative Syndrome Scale (PANSS). Potential cognitive dysfunctions in patients may also reduce the validity of responses on self-report measures. Therefore, in future, we recommend evaluating psychiatric symptoms and cognitive functions using PANSS, BPRS, and the Brief Assessment of Cognition in Schizophrenia, and conducting a comprehensive analysis including psychiatric symptoms and cognitive functions. Finally, this study investigated the association between self-stigma, depressive symptoms, autistic symptoms, and recovery combining outpatients and inpatients using SEM analysis. This study found a significant difference between outpatients and inpatients only in terms of the AQ subscale “attention to detail”. Although bivariate correlational analysis in this study showed that only “attention to detail” was not related to self-stigma, depressive symptoms, and recovery in both inpatients and outpatients (see additional files 1 and 2), hospitalization can affect patient psychopathology. Therefore, the relationship between self-stigma, autism symptoms, depressive symptoms, and recovery needs to be analyzed in terms of outpatients and inpatients separately using a larger sample size.

## Conclusions

The study is the first to show the mediating effects of self-stigma and depression on the association between autistic symptoms and recovery, as well as the direct effect of stigma resistance on recovery in patients with schizophrenia-spectrum disorders. The results of this study may provide potentially meaningful insight into the psychological structure of recovery and could help inform the development of effective interventions to achieve recovery in patients with schizophrenia-spectrum disorders. Because this study had a cross-sectional study design, further longitudinal studies may be needed to identify causal relationships between self-stigma, autistic and depressive symptoms, and recovery.

## Supplementary Information

**Additional file 1.**

**Additional file 2.**

## Data Availability

The data that support the findings of this study are available from the corresponding author, HK, upon reasonable request.

## Abbreviations

AGFI: adjusted goodness-of-fit index
AIC: Akaike information criterion
AQ: Autism Spectrum Quotient
AQ-J: Autism-Spectrum Quotient, Japanese version
CFI: comparative fit index
GFI: goodness-of-fit index
ISMI: Internalized Stigma of Mental Illness Scale
ISMI-J: Internalized Stigma of Mental Illness Scale, Japanese version
QIDS: Quick Inventory of Depressive Symptomatology
QIDS-J: Quick Inventory of Depressive Symptomatology, Japanese version
QOL: quality of life
RAS: Recovery Assessment Scale
RAS-J: Recovery Assessment Scale, Japanese version
RMSEA: root mean square error of approximation
SEM: structural equation modeling
SDE: standardized direct effect
SIDE: standardized indirect effect
